# Monocytes, Peripheral Blood Mononuclear Cells, and THP-1 Cells Exhibit Different Cytokine Expression Patterns following Stimulation with Lipopolysaccharide

**DOI:** 10.1155/2013/697972

**Published:** 2013-05-29

**Authors:** Anita Schildberger, Eva Rossmanith, Tanja Eichhorn, Katharina Strassl, Viktoria Weber

**Affiliations:** ^1^Department for Health Sciences and Biomedicine, Center for Biomedical Technology, Danube University Krems, Dr.-Karl-Dorrek-Straße 30, 3500 Krems, Austria; ^2^Christian Doppler Laboratory for Innovative Therapy Approaches in Sepsis, Danube University Krems, Dr.-Karl-Dorrek-Straße 30, 3500 Krems, Austria

## Abstract

THP-1 cells are widely applied to mimic monocytes in cell culture models. In this study, we compared the cytokine release from THP-1, peripheral blood mononuclear cells (PBMC), monocytes, or whole blood after stimulation with lipopolysaccharide (LPS) and investigated the consequences of different cytokine profiles on human umbilical vein endothelial cell (HUVEC) activation. While *Pseudomonas aeruginosa*-stimulated (10 ng/mL) THP-1 secreted similar amounts of tumor necrosis factor alpha (TNF-**α**) as monocytes and PBMC, they produced lower amounts of interleukin(IL)-8 and no IL-6 and IL-10. Whole blood required a higher concentration of *Pseudomonas aeruginosa* (1000 ng/mL) to induce cytokine release than isolated monocytes or PBMC (10 ng/mL). HUVEC secreted more IL-6 and IL-8 after stimulation with conditioned medium derived from whole blood than from THP-1, despite equal concentrations of TNF-**α** in both media. Specific adsorption of TNF-**α** or selective cytokine adsorption from the conditioned media prior to HUVEC stimulation significantly reduced HUVEC activation. Our findings show that THP-1 differ from monocytes, PBMC, and whole blood with respect to cytokine release after stimulation with LPS. Additionally, we could demonstrate that adsorption of inflammatory mediators results in reduced endothelial activation, which supports the concept of extracorporeal mediator modulation as supportive therapy for sepsis.

## 1. Introduction

As a barrier between the blood stream and the surrounding tissues, the endothelium is involved in regulation of blood flow, vascular tone, thrombosis, thrombolysis, adherence of platelets, and extravasation of circulating leukocytes [1, 2]. In infection, endothelial cells are activated either directly by pathogen-associated molecular patterns, such as lipopolysaccharide (LPS) from Gram-negative bacteria, or by host-derived mediators, such as chemokines, cytokines, complement, and serine proteases [3, 4]. The endothelium responds to these mediators by switching to a proinflammatory and procoagulant state, which is associated with enhanced adhesion of platelets, monocytes, and neutrophils. Endothelial injury and endothelial dysfunction are involved in a variety of disease processes, including atherosclerosis, inflammatory syndromes, sepsis, and multiple organ failure [5].

Sepsis and sepsis-associated multiple organ failure arise in response to severe microbial infection with extensive tissue damage due to overactivation of the innate immune system and the proinflammatory cascade [6, 7]. Worldwide, sepsis is one of the leading causes of morbidity and mortality, and its incidence continues to increase [8]. The development of targeted therapies for sepsis remains a major challenge due to the extreme heterogeneity of septic patients and due to the complex network of inflammatory mediators involved in the septic process. Numerous clinical trials using specific antagonists, such as antibodies or soluble receptor constructs, to target individual inflammatory mediators were performed in the last two decades, but none of these trials did result in convincingly improved survival rates [9]. Extracorporeal blood purification techniques, such as hemofiltration or apheresis, have been proposed as possible strategies to modulate the multiple inflammatory mediators in sepsis. A potential advantage of extracorporeal approaches is that they affect only excess circulating pools of inflammatory mediators, while systemic administration of specific antagonists leads to a complete blockade of their targets also in tissues [10, 11], which may actually be detrimental. 

To assess the effect of mediator modulation on endothelial activation and thus to support the preclinical development of extracorporeal adsorption therapies, we have previously established a cell culture model based on stimulation of monocytic THP-1 cells with lipopolysaccharide (LPS) in media containing human plasma. Culture supernatants derived from the stimulated THP-1 cells containing LPS and mediators secreted by THP-1 cells in response to stimulation were used to activate human umbilical vein endothelial cells (HUVEC). This model allows assessing the effect of mediator modulation with adsorbent polymers on subsequent endothelial activation [12, 13].

Due to their uniform genetic background, THP-1 cells are frequently used as a model system for monocytes. They have been shown to respond with a similar transcriptional pattern as PBMC-derived macrophages after stimulation with LPS from *E. coli* [14]. Since the comparability of LPS-induced cytokine secretion between THP-1 cells and PBMCs or monocytes is crucial for all cell culture models employing these cell types, we aimed to compare the LPS-induced cytokine secretion patterns of THP-1 cells, PBMC, monocytes, or whole blood and to assess their influence on subsequent activation of endothelial cells. 

## 2. Materials and Methods

### 2.1. Cell Culture Media and Reagents

Medium 199 (M199), RPMI-1640 (RPMI), phosphate-buffered saline (PBS), bovine serum albumin (BSA), ethylene diamine tetraacetic acid disodium salt (EDTA), 4-(2-hydroxyethyl)-1-piperazineethanesulfonic acid (HEPES), penicillin-streptomycin (PS), and LPS from *P. aeruginosa* or *E. coli* (O55:B5) were purchased from Sigma-Aldrich (St Louis, MO, USA). Fetal bovine serum (FBS) was obtained from PAA Laboratories GmbH (Pasching, Austria). Heparin (5000 IU/mL) was from Baxter (Vienna, Austria).

### 2.2. Blood and Plasma

Blood was freshly drawn from healthy volunteers after informed written consent and anticoagulated with sodium citrate at a final concentration of 12.9 mM (Mayrhofer Pharmazeutika, Leonding, Austria). Human plasma was obtained from a local plasma donation center.

### 2.3. Cells and Cell Culture

The human monocytic cell line THP-1 was obtained from the American Type Culture Collection (Nr. TIB-202) and was maintained as described in [12]. Isolation of PBMC from human blood was performed by density gradient centrifugation on Ficoll-Paque PLUS (GE Healthcare, Uppsala, Sweden). Briefly, 300 mL of freshly drawn blood was mixed at a ratio of 1 : 1 (vol/vol) with PBS at room temperature and 30 mL of the mixture was layered to 15 mL Ficoll-Paque PLUS in 50 mL tubes (NALGENE polycarbonate round-bottom tubes, Thermo Fisher Scientific, Waltham, MA, USA). The tubes were pretreated with Sigmacote (Sigma-Aldrich, St Louis, MO, USA) and sterilized before use. After centrifugation at 1180 g for 30 min at 20°C without break, buffy coats were collected, pooled, resuspended in PBS and centrifuged at 1180 g for 20 min at 20°C without break. The pellets were resuspended in 60 mL of PBS and again layered onto Ficoll-Paque PLUS as described previously. After centrifugation at 160 g for 20 min at 20°C without break, the top 15 mL of platelet-rich solution was discarded and the rest was recentrifuged at 350 g for 20 min at 20°C without break. Buffy coats were aspirated and washed with ice-cold PBS containing 0.1 wt% BSA and 2 mM EDTA and centrifuging at 400 g for 8 min at 4°C without break. One half of the harvested PBMCs was cultured overnight in RPMI-1640 supplemented with 0.02 M HEPES, 100 *μ*M PS, and 10 vol% heat-inactivated FBS in HydroCell Surface 24-well plates (Thermo Fisher Scientific, Waltham, Massachusetts, USA) in humidified atmosphere (5 vol% CO_2_, 37°C). The other half of the harvested PBMCs was used to isolate monocytes using the Dynabeads Untouched Human Monocytes Kit (Invitrogen, Lofer, Austria) according to the manufacturer's protocol. Isolated monocytes were cultured overnight under the same conditions as PBMCs. Isolation and maintenance of primary HUVEC were performed as described before [12].

### 2.4. Stimulation of Monocytes, PBMC, or THP-1 with LPS

**Figure 1:**
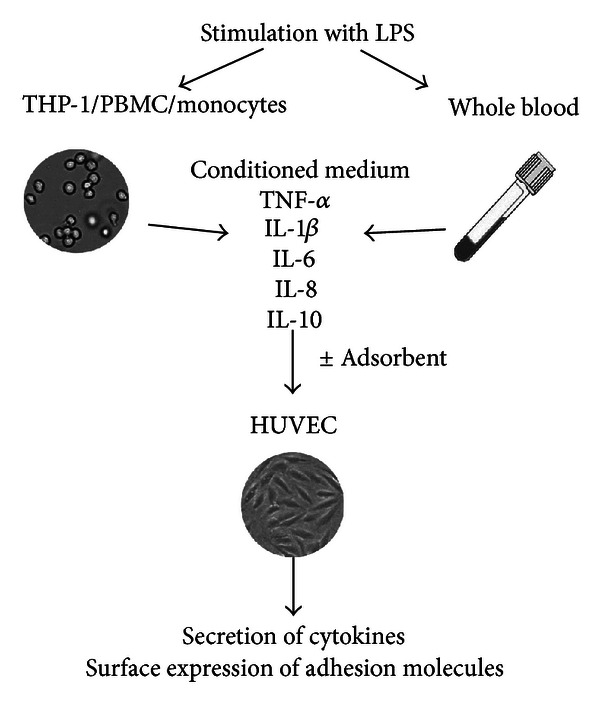
Scheme of the cell culture model. THP-1 cells, peripheral blood mononuclear cells (PBMC), monocytes, or whole blood are stimulated with lipopolysaccharide (LPS). The harvested conditioned medium containing LPS and cytokines is treated with adsorbents to modulate mediators of inflammation and is subsequently used to stimulate human umbilical vein endothelial cells (HUVEC).

Cells were suspended in M199 containing 0.02 M HEPES, 100 *μ*M penicillin-streptomycin, 6 IU/mL heparin, and 10 vol% human plasma at a concentration of 1 × 10^6^ cells/mL and stimulated with 10 ng/mL LPS from *P. aeruginosa*. Stimulations were carried out in HydroCell Surface 24-well plates (1 mL/well) for 1, 2, 4, 6, 8, and 24 h in humidified atmosphere (5 vol% CO_2_, 37°C). Afterwards, the cell suspensions were pelleted by centrifugation, the supernatants (conditioned media) were harvested, aliquoted, and stored at −70°C until quantification of cytokines or HUVEC stimulation (Figure 1). 

### 2.5. Stimulation of HUVEC with Conditioned Media from Monocytes, PBMC, or THP-1

Endothelial cells were suspended in M199 supplemented with 0.02 M HEPES, 100 *μ*M penicillin streptomycin, 6 IU/mL heparin, 10 *μ*g/mL ECGS, and 20 vol% FBS and seeded into 96-well plates at a density of 18000 cells/well (Greiner Bio-One, Kremsmünster, Austria). After an overnight incubation, HUVEC were washed with M199 and stimulated with conditioned media which were derived from a 4, 8, or 24 h stimulation of THP-1, PMBC, or monocytes. HUVEC stimulation was performed using 200 *μ*L of conditioned media per well for 16 h in a humidified atmosphere. Thereafter, supernatants were aspirated, centrifuged at 15500 g (5 min, 4°C), and stored at −70°C until quantification of cytokines.

### 2.6. Stimulation of Whole Blood with LPS

Aliquots of 1.5 mL of freshly drawn heparin-anticoagulated human blood (5 IU/mL blood) from healthy volunteers were spiked with either 10 or 1000 ng/mL LPS from *P. aeruginosa* or *E. coli*. Samples without LPS served as control. After stimulation for 1, 2, 4, 6, 8, or 24 h at 37°C with gentle shaking, the samples were centrifuged at 1500 g (5 min, 4°C), and the plasma was stored at −70°C until further use.

### 2.7. Adsorption of Mediators of Inflammation

Blood was stimulated with 100 ng/mL LPS from *E. coli* for 4 h as described previously. The plasma resulting from this stimulation was diluted tenfold with M199 to obtain conditioned medium derived from blood (CMB). THP-1 cells were stimulated with 10 ng/mL LPS from *E. coli* for 4 h as described previously to obtain conditioned medium derived from THP-1 cells (CMT). An adsorbent for the specific binding of TNF-*α* was prepared by covalent binding of a chimeric human-mouse monoclonal anti-TNF-*α* antibody (Infliximab, Centocor, Leiden, The Netherlands) onto cyanogen bromide-activated Sepharose 4B (GE Healthcare, Uppsala, Sweden). A polystyrene-divinylbenzene (PS-DVB) copolymer (Amberchrom HPR10; Dow Chemical, Midland, MI, USA) was used as a selective adsorbent to bind cytokines and complement factors [15–17]. The PS-DVB copolymer was coated with human serum albumin (Octapharma, Vienna, Austria) to improve its blood compatibility. The conditioned media derived from blood or THP-1 stimulation were incubated with the adsorbents for 1 h at 37°C with gentle shaking. A ratio of 1 vol% (TNF-*α* adsorbent) or 10 vol% (PS-DVB) of adsorbent to medium was used. After treatment with the adsorbents, aliquots of 1.5 mL of the media were applied to HUVEC (400000 cells per well of a 6-well culture plate) for 15 h in humidified atmosphere (5 vol% CO_2_, 37°C). Thereafter, culture supernatants were aspirated, centrifuged at 15500 g for 5 min at 4°C, aliquoted, and stored at −70°C until quantification of cytokines. Surface expression of the adhesion molecules E-selectin and ICAM-1 was determined with flow cytometry.

### 2.8. Quantification of Cytokines

Concentrations of TNF-*α*, IL-1*β*, IL-6, IL-8, and IL-10 were determined using the Bio-Plex 200 system (Bio-Rad, Vienna, Austria).

### 2.9. Flow Cytometric Analysis

The purity of monocytes was assessed by determining the CD14-positive cell population. After monocyte isolation, cells were washed twice with ice-cold PBS and stained with FITC-conjugated anti-CD14 or with the respective IgG control antibody (Becton Dickinson, Vienna, Austria) in PBS supplemented with 2 vol% FBS on ice for 30 min. After two washing steps, cells were analyzed on a FACScan flow cytometer and data were analyzed using the CellQuest software (Becton Dickinson, Vienna, Austria). For detection of the surface expression of the adhesion molecules E-selectin and ICAM-1, HUVEC were detached using 0.02 wt% EDTA and washed with ice-cold PBS containing 0.1 wt% sodium azide (Sigma-Aldrich, St Louis, MO, USA). Staining of surface markers was performed by incubation with PE-conjugated anti-E-selectin, PE-Cy5-conjugated anti-ICAM-1, or the respective IgG control antibodies (Becton Dickinson, Vienna, Austria) in PBS supplemented with 2 vol% FBS on ice for 30 min. After two washing steps, 10000 gated cells were analyzed on a FC 500 flow cytometer and data were analyzed using the CXP software (Beckman Coulter, Vienna, Austria).

### 2.10. Statistical Analysis

Statistical analysis was performed using the software package SPSS Statistics for Windows, version 18.0 (SPSS Inc., Chicago, IL, USA). When comparing two groups, data were analysed by the nonparametric Wilcoxon rank sum test. Data are expressed as means ± SD. Significance was accepted at *P* ≤ 0.05. 

## 3. Results

### 3.1. Cytokine Release upon Stimulation of Monocytes, PBMC, and THP-1 Cells with LPS

**Figure 2:**
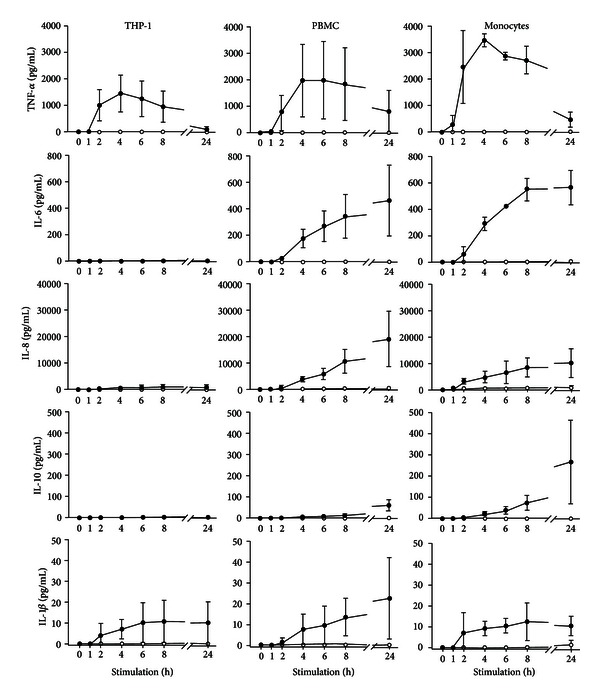
Cytokine release upon stimulation of THP-1, PBMC, and monocytes with lipopolysaccharide. Cultures of one million cells per mL of medium containing 10 vol% plasma were stimulated with 10 ng per mL of LPS from *P. aeruginosa* (black circles). Unstimulated cultures served as control (white circles). Concentrations of TNF-*α*, IL-6, IL-8, IL-10, and IL-1beta are given as mean ± SD (*n* = 3).

Monocytes were 95% pure according to flow cytometry. The stimulation of THP-1 cells, PMBC, and monocytes with LPS resulted in comparable TNF-*α* expression patterns with a peak at 4 h and a subsequent decrease over time (Figure 2). Monocytes secreted higher amounts of TNF-*α* than PBMC and THP-1 cells (3460 ± 245 pg/mL versus 1960 ± 1365 pg/mL versus 1440 ± 696 pg/mL at 4 h, resp.). After 24 h of stimulation, TNF-*α* levels declined to 460 pg/mL (monocytes), 790 pg/mL (PBMC), and 110 pg/mL (THP-1 cells), respectively. THP-1 cells did not secrete IL-6 and IL-10, while PBMC and monocytes produced increasing amounts of IL-6 (460 ± 270 pg/mL and 560 ± 130 pg/mL after 24 h) and IL-10 (60 ± 30 pg/mL and 270 ± 200 pg/mL after 24 h) over time. THP-1 cells secreted 900 ± 830 pg/mL of IL-8 after 24 h of stimulation, whereas PBMC and monocytes exhibited much higher IL-8 release (18970  ±  10590 pg/mL and 10100  ±  5400 pg/mL). In summary, monocytes and PBMC secreted comparable amounts of TNF-*α*, IL-6, IL-8, and IL-10 over time. THP-1 showed a secretion of TNF-*α* that was comparable to monocytes and PBMC but released by far less IL-8 than PBMC or monocytes and failed to secrete IL-6 and IL-10. 

### 3.2. Stimulation of HUVEC with Conditioned Media

**Figure 3:**
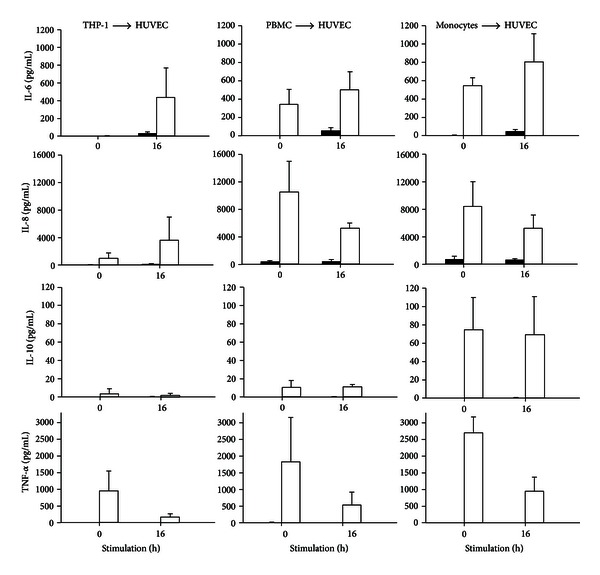
Effect of conditioned media on HUVEC. Conditioned media (white bars) derived from an 8 h stimulation of THP-1, PBMC, or monocytes with LPS were applied onto HUVEC and the cytokine release was measured. Concentrations of IL-6, IL-8, IL-10 and TNF-*α* are expressed as mean ± SD (*n* = 3). Black bars indicate control media without LPS.

Conditioned media derived from THP-1 cells, PBMC, or monocytes after 4, 8, or 24 h of stimulation with LPS were used to stimulate HUVEC for 16 h (Figure 3, values for 8 h are shown). HUVEC secreted less IL-6 with conditioned media derived from PBMC and monocytes than with conditioned media derived from THP-1 cells. IL-8 levels increased after HUVEC stimulation with conditioned media from THP-1 cells but decreased after stimulation with conditioned media from PBMC and monocytes. For all three conditioned media, IL-10 concentrations remained stable after HUVEC stimulation, while TNF-*α* concentrations decreased. 

### 3.3. Stimulation of Whole Blood with LPS

**Figure 4:**
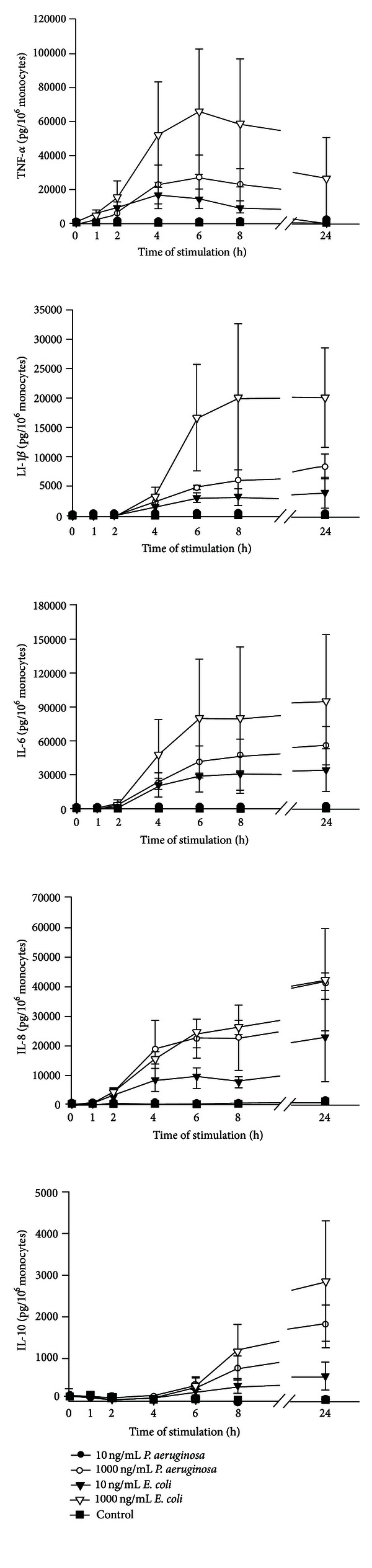
Effect of LPS on cytokine secretion from whole blood. Freshly drawn blood was stimulated with 10 or 1000 ng/mL LPS from *P. aeruginosa* or *E. coli*. Samples without LPS served as control. Concentrations of TNF-*α*, IL-1*β*, IL-6, IL-8, and IL-10 are expressed as mean ± SD (*n* = 3).

In addition to THP-1 cells, PBMC, or monocytes, whole blood was stimulated with LPS. Freshly drawn blood anticoagulated with heparin (5 IU/mL blood) was treated with LPS for up to 24 h. In contrast to the stimulation of THP-1 cells, PBMC, or monocytes, 10 ng/mL LPS from *P. aeruginosa* did not lead to cytokine secretion from whole blood (Figure 4). However, 1000 ng/mL LPS from *P. aeruginosa* lead to significant secretion of TNF-*α*, IL-1*β*, IL-6, IL-8, and IL-10. LPS from *E. coli* showed a higher stimulatory potential than *P. aeruginosa*, and 10 ng/mL was sufficient to elicit secretion of TNF-*α*, IL-1*β*, IL-6, IL-8, and IL-10.

### 3.4. Effect of Cytokine Adsorption on HUVEC Activation

**Figure 5:**
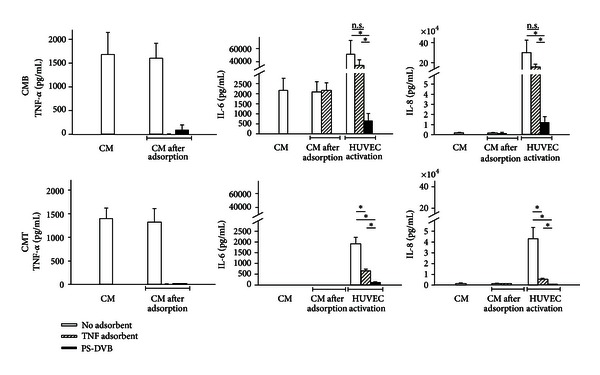
Effect of mediator modulation on HUVEC activation. Whole blood was stimulated with 100 ng/mL LPS from *E. coli* for 4 h and diluted tenfold with M199 to obtain conditioned medium derived from blood (CMB). THP-1 cells were stimulated with 10 ng/mL LPS from *E. coli* for 4 h in medium M199 supplemented with 10 vol% human plasma to yield conditioned medium derived from THP-1 cells (CMT). HUVEC were stimulated with CMB and CMT, respectively. Concentrations of TNF-*α*, IL-6 and IL-8 prior to mediator modulation (CM) and after specific TNF-*α* adsorption or selective cytokine adsorption (PS-DVB) are shown. Data are given as mean of three experiments ± standard deviation. Significance was accepted at *P* ≤ 0.05. n.s.: not significant.

**Figure 6:**
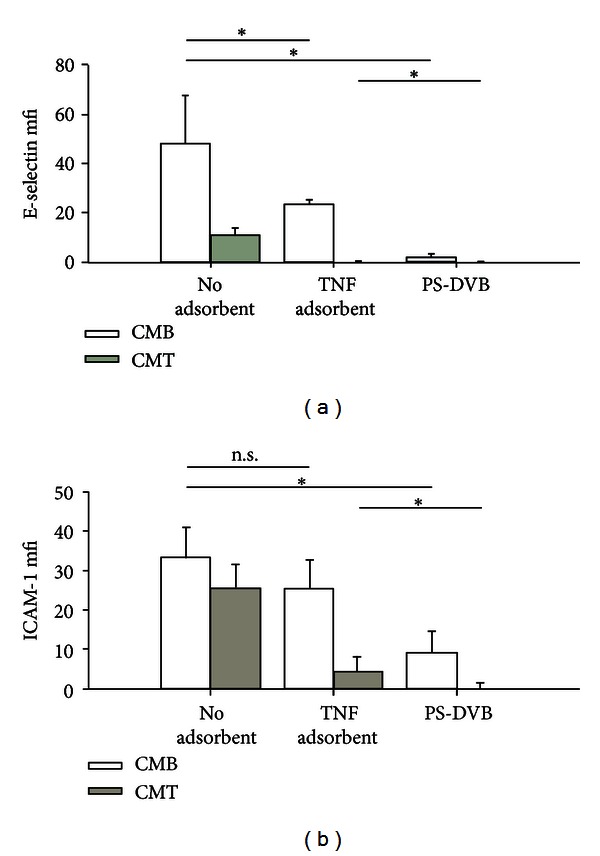
Effect of mediator modulation on adhesion molecule expression. The experimental setup was identical to Figure 5. HUVEC surface expression of the adhesion molecules E-selectin (a) and ICAM-1 (b) is expressed as mean fluorescent intensity (mfi) minus basal expression of adhesion molecules on HUVEC. Data are given as mean of three experiments ± standard deviation. Significance was accepted at *P* ≤ 0.05. n.s.: not significant.

Given the different cytokine expression patterns of THP-1 cells and whole blood, we aimed to investigate whether these differences would also have an effect on subsequent HUVEC activation. Therefore, whole blood was stimulated with 100 ng/mL LPS from *E. coli* for 4 h and the plasma was diluted tenfold with M199 to obtain conditioned medium derived from blood (CMB), which contained 1650 ± 450 pg/mL TNF-*α*, 2000 ± 600 pg/mL IL-6, and 1500 ± 300 pg/mL IL-8 (Figure 5). In parallel, THP-1 cells were stimulated with 10 ng/mL LPS from *E. coli* for 4 h in medium M199 supplemented with 10 vol% plasma to yield conditioned medium derived from THP-1 cells (CMT) which contained 1400 ± 200 pg/mL TNF-*α* but no IL-6 and lower concentrations of IL-8 (900 ± 100 pg/mL). The conditioned media were treated with either a specific adsorbent for TNF-*α* or with a selective cytokine adsorbent. Treatment with the specific TNF-*α* adsorbent resulted in complete removal of TNF-*α* from CMB and CMT. The selective cytokine adsorbent completely removed IL-6 and IL-8 and decreased TNF-*α* concentrations by 95%. After specific adsorption of TNF-*α* or selective cytokine adsorption from CMB or CMT, HUVEC were stimulated with the conditioned media and the secretion of IL-6 and IL-8 and surface expression of the adhesion molecules E-selectin and ICAM-1 were monitored. Secretion of IL-6 (52000 ± 22000 versus 2000 ± 400 pg/mL) and IL-8 (295000 ± 128000 versus 43000  ±  11000 pg/mL) was higher for CMB than for CMT despite equal TNF-*α* concentrations in both media (Figure 5), indicating the presence of additional stimulatory factors in CMB next to TNF-*α*. Pretreatment of conditioned media with the specific TNF-*α* adsorbent or the selective cytokine adsorbent resulted in decreased secretion of IL-6 and IL-8 from HUVEC. For conditioned medium derived from blood, the release of IL-6 was reduced to 63% (not significant) and 1% (*P* < 0.05) for specific TNF-*α* adsorption and selective cytokine adsorption, respectively, while IL-8 release was reduced to 54% and 4% (*P* < 0.05 in both cases). For conditioned medium derived from THP-1 cells, the release of IL-6 was reduced to 33% (not significant) and 6% (*P* < 0.05), and IL-8 release was reduced to 12% (*P* < 0.05) and 2% (*P* < 0.05) for specific adsorption of TNF-*α* versus selective cytokine adsorption. Thus, selective cytokine adsorption had a much stronger influence on HUVEC activation as compared to specific TNF-*α* adsorption. Regarding the expression of surface adhesion molecules, HUVEC exhibited significantly higher expression of E-selectin after stimulation with conditioned medium derived from blood as compared to conditioned medium derived from THP-1 cells. Adsorption of TNF-*α* resulted in significantly decreased or completely abolished E-selectin expression for CMB and CMT, respectively. Selective cytokine adsorption completely abolished E-selectin expression with both stimulation media (Figure 6). 

## 4. Discussion

Extracorporeal modulation of inflammatory mediators, such as cytokines, with filters or adsorbents is regarded as a promising supportive therapy for sepsis. During preclinical development of such extracorporeal approaches, cell culture models allow to assess the biological effect of mediator modulation. We have previously established a cell culture model based on stimulation of monocytic THP-1 cells with lipopolysaccharide and subsequent activation of endothelial cells with the conditioned medium [12]. THP-1 cells are widely used to study the function of monocytes [18]. One of their major advantages over primary monocytes is their homogenous genetic background, which abolishes donor variability. Further, they are easily accessible and can be obtained without contamination with other blood components, while the availability of primary human monocytes is limited. In several studies, THP-1 have been shown to represent a more mature monocytic phenotype than other immortalized human monocyte cell lines, such as U937 cells [19], and it has been demonstrated that the interaction between THP-1 and endothelial cells is comparable to human primary monocytes [20–22]. Gene expression profiles of THP-1 after LPS stimulation are very similar to primary PBMC-derived macrophages [14]. Still, the extent to which THP-1 cells mimic monocytes is not fully elucidated, which prompted us to compare THP-1 cells to freshly isolated human peripheral blood mononuclear cells (PBMC) or monocytes. We found that upon stimulation with LPS, primary human monocytes and PBMC secreted comparable amounts of TNF-*α*, IL-6, IL-8, and IL-10 over time, while THP-1 cells secreted similar amounts of TNF-*α* but did not secrete IL-6 and IL-10. Moreover, their release of IL-8 was much lower than the observed one for primary human monocytes and PBMC under identical experimental conditions. 

The expression pattern of TNF-*α* was in accordance with previously published data showing that monocytes reacted to an LPS stimulus by secretion of TNF-*α in vivo* or *in vitro* within the first hours after stimulation and that TNF-*α* concentration declined after the initial peak [23]. In contrast to our findings, THP-1 cells have been reported in the literature to secrete IL-6 and IL-10 [24, 25], albeit under different experimental conditions, as THP-1 cells were differentiated to macrophages and higher LPS concentrations were used in the published studies.

In addition to monocytes, PBMC, and THP-1, we stimulated whole blood with lipopolysaccharide. During whole blood stimulation, cells are not stressed by isolation and cultivation procedures. In addition, whole blood stimulation experiments are faster and cheaper to perform. In our study, a concentration of 10 ng/mL LPS from *P. aeruginosa* was sufficient to stimulate isolated cells, while 1000 ng/mL was needed to activate whole blood. In accordance with literature [26], lipopolysaccharide from *E. coli* showed a higher stimulatory potential and 10 ng/mL elicited cytokine secretion from whole blood. Thus, isolated blood cells show higher sensitivity to stimulation with lipopolysaccharide as compared to whole blood, which may be due to interaction of LPS with other blood components, such as lipoproteins. 

As shown previously, endothelial cells react to culture supernatants from LPS-activated THP-1 cells by increased gene expression of inflammation-related factors [27], by increased activity of NF-*κ*B, by increased secretion of cytokines and plasminogen activator inhibitor, and by enhanced surface expression of adhesion molecules such as ICAM-1 and E-selectin. In this study, we chose secretion of IL-6 and IL-8 as activation markers and showed that conditioned media derived from PBMC and monocytes resulted in comparable IL-6 secretion from HUVEC, whereas no IL-8 was secreted, in contrast to the use of conditioned media from THP-1 cells. Conditioned medium derived from whole blood stimulation activated HUVEC even to a higher extent than conditioned medium derived from THP-1 cells despite comparable concentrations of LPS and TNF-*α* in both media. Mediator modulation with either a specific adsorbent for TNF-*α* or with a selective polystyrene divinylbenzene copolymer, which binds to a range of cytokines, significantly reduced subsequent HUVEC activation. While the specific adsorbent resulted in reduction of HUVEC cytokine release to at least 50%, the effect of selective cytokine adsorption was even more pronounced with a reduction of cytokine secretion by more than 90%, indicating that factors in addition to TNF-*α* are relevant for HUVEC stimulation. These findings support the concept of selective mediator modulation as supportive therapy for sepsis rather than the specific targeting of individual factors.
